# Individualized treatment of diabetes mellitus in older adults

**DOI:** 10.1111/ggi.14979

**Published:** 2024-10-07

**Authors:** Atsushi Araki

**Affiliations:** ^1^ Department of Diabetes, Metabolism, and Endocrinology Tokyo Metropolitan Institute for Geriatrics and Gerontology Tokyo Japan; ^2^ Center for Comprehensive Care and Research for Prefrailty Tokyo Metropolitan Institute for Geriatrics and Gerontology Tokyo Japan

**Keywords:** comprehensive, diabetes mellitus, geriatric syndromes, multimorbidity, older adults

## Abstract

The population of older adults with diabetes mellitus is growing but heterogeneous. Because geriatric syndromes, comorbidity or multimorbidity, the complexity of glucose dynamics, and socioeconomic conditions are associated with the risk of severe hypoglycemia and mortality, these factors should be considered in individualized diabetes treatment. Because cognitive impairment and frailty have similar etiologies and risk factors, a common strategy can be implemented to address them through optimal glycemic control, management of vascular risk factors, diet, exercise, social participation, and support. To prevent frailty or sarcopenia, optimal energy intake, adequate protein and vitamin intake, and resistance or multi‐component exercise are recommended. For hypoglycemic drug therapy, it is important to reduce hypoglycemia, to use sodium glucose cotransporter‐2 (SGLT2) inhibitors and glucagon‐like peptide‐1 (GLP‐1) receptor agonists, taking into account the benefits for cardiovascular disease and the risk of adverse effects, and to simplify treatment to address poor adherence. Glycemic control goals for older adults with diabetes should be set according to three categories, based on cognitive function and activities of daily living, using the Dementia Assessment Sheet for Community‐based Integrated Care System 8‐items. This categorization can be used to determine treatment strategies for diabetes when combined with the Comprehensive Geriatric Assessment (CGA). Based on the CGA, frailty prevention, treatment simplification, and social participation or services should be implemented for patients in Category II and above. Measures against hypoglycemia and for the prevention of cardiovascular disease and chronic kidney disease should also be promoted. Treatment based on categorization and CGA by multidisciplinary professionals would be an individualized treatment for older adults with diabetes. **Geriatr Gerontol Int 2024; 24: 1257–1268**.

## Graphical abstract

Geriatric syndromes, multimorbidity, complexity of glycemic dynamics, and socioeconomic conditions should be considered in individualized diabetes management because of the risk of hypoglycemia and mortality. Treatment based on categorization using the Dementia Assessment Sheet for Community‐based Integrated Care System 8‐items and Comprehensive Geriatric Assessment by multidisciplinary professionals would be an individualized treatment for older adults with diabetes.

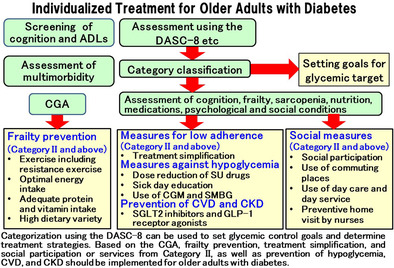

## Introduction

**Figure 1:**
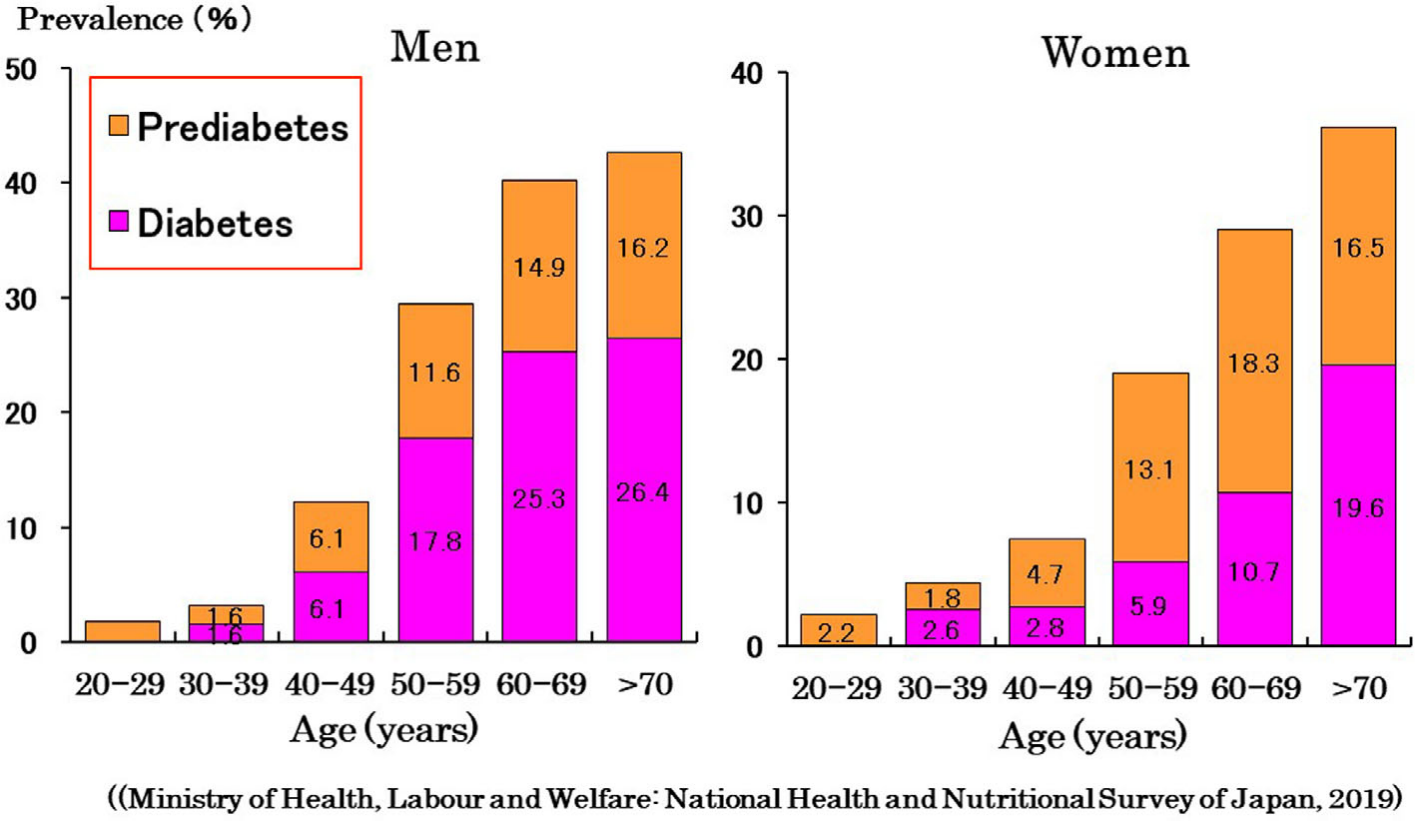
Diabetes and prediabetes increase with age.

The number of adults with diabetes mellitus was estimated to be 537 million worldwide in 2021, and is anticipated to increase to 700.2 million by 2045.^1^ This is due to general population aging, as well as to lifestyle changes. According to the National Health and Nutrition Survey in Japan, 26.4% of men and 19.6% of women >70 years of age have diabetes (Fig. 1).^2^ Based on the population in 2021, 3.94 million and 4.01 million people aged 65–74 years and ≥75 years, respectively, in Japan have diabetes—with the number of patients with diabetes in the old‐old group being significantly greater than that in the young‐old one.

Older adults with diabetes are generally defined as those aged ≥65 years. However, diabetes in adults aged ≥75 years differs from that in those aged 65–74 years in terms of physical, mental, and social aspects. That is, patients with diabetes aged ≥75 years are likely to have more geriatric syndromes, such as cognitive impairment and frailty, as well as comorbidities, such as heart failure and severe hypoglycemia. They are also much more likely to require social support. Therefore, diabetes patients aged >75 years should be treated as older diabetes, which requires particular attention.

**Figure 2:**
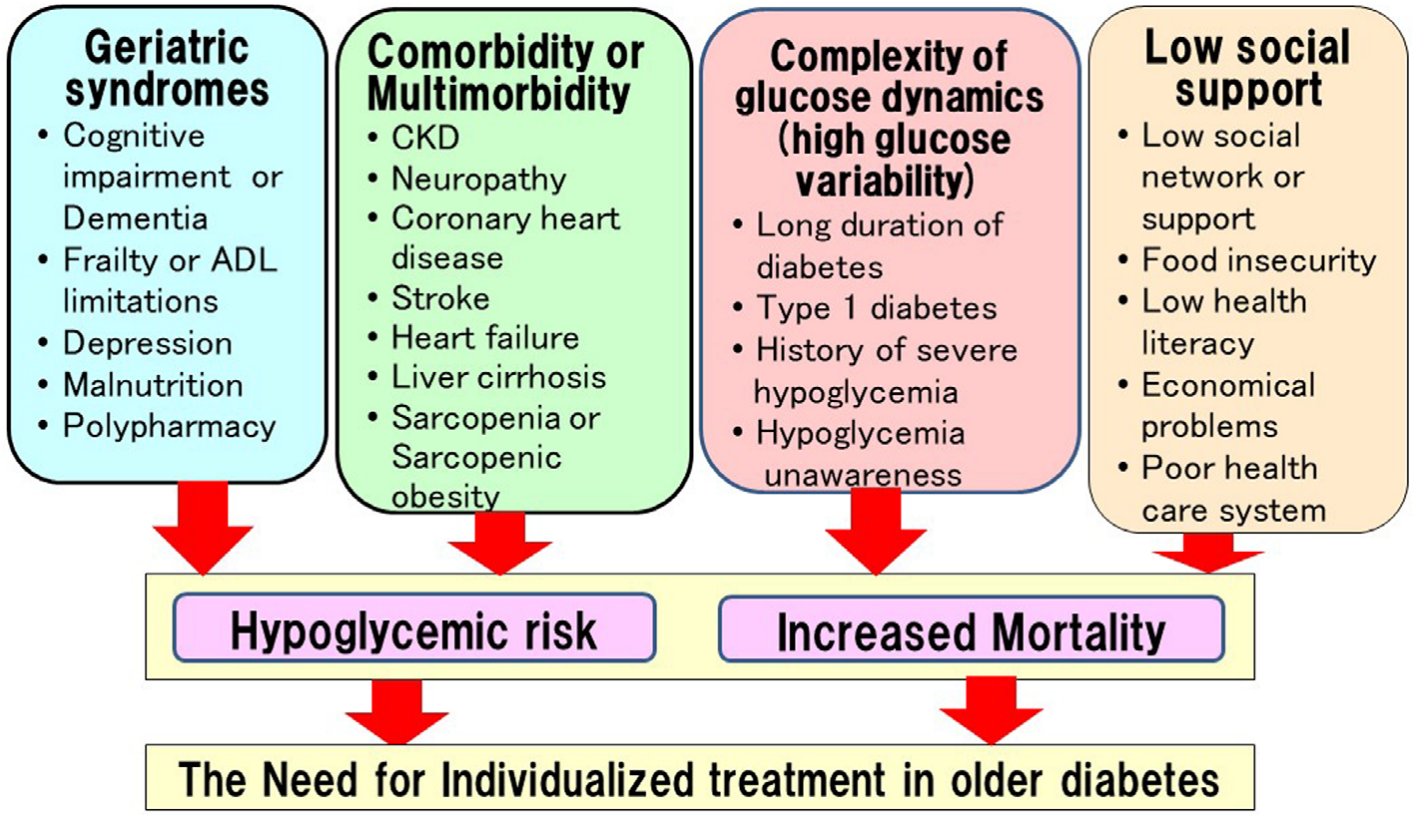
Factors for consideration in individualized treatment for older adults with diabetes. (i) Geriatric syndromes, (ii) comorbidities and multimorbidities, (iii) complexities of glucose dynamics (i.e., high glucose variability), and (iv) socioeconomic conditions are all associated with the risk of severe hypoglycemia and mortality. Therefore, these four factors should be considered in the individualized treatment of older adults with diabetes.

Diabetes in older adults is not uniform; rather, it is characterized by significant differences among individuals. Factors that increase variability in older adults with diabetes include (i) geriatric syndromes, (ii) comorbidities and multimorbidities, (iii) complex glucose dynamics (i.e., high glucose variability), and (iv) socioeconomic conditions (Fig. 2). Geriatric syndromes,^3^ multimorbidity,^4^ increased glucose variability,^5^, ^6^ and lack of social support^7^, ^8^ are all associated with the risk of severe hypoglycemia and mortality. Therefore, individualized treatment for older adults with diabetes should consider these four factors. This paper focuses on the individualized treatment of diabetes in older adults.

## Diabetes and geriatric syndromes

Older adults with diabetes are prone to geriatric syndromes, such as cognitive impairment, limitations in activities of daily living (ADL), frailty, falls, depressive symptoms, malnutrition, and polypharmacy.^3^ Hyperglycemia predisposes to geriatric syndromes, whereas hypoglycemia is a risk factor for some geriatric syndromes such as dementia, depression, falls, and fractures.^3^

### Diabetes and cognitive impairment or dementia

A growing body of evidence supports the association between diabetes and cognitive impairment. A meta‐analysis of 114 prospective studies found that patients with diabetes were 1.5 times more likely to have Alzheimer's disease, 2 times more likely to have vascular dementia, and 1.5 times more likely to have mild cognitive impairment (MCI).^9^ Regarding cognitive function, global cognitive and executive functions and memory are likely to be impaired, which may contribute to poor adherence to diabetic self‐care.^9^ Hyperglycemia represents a risk factor for dementia, with longitudinal studies reporting that diabetes status, poor glycemic control (HbA1c ≥ 7%), and longer diabetes duration are all associated with incident cognitive impairment, and that an HbA1c of ≥8.0% nearly doubles the risk of developing MCI and dementia.^10^ A prospective study of 461 563 participants from the UK Biobank showed that a combination of poor glycemic control (HbA1c ≥ 8%) and long duration of diabetes (≥10 years) was associated with the highest risk of all‐cause dementia.^11^ The population of patients with untreated diabetes is 1.60 times more likely to develop dementia, and to have higher total tau and β‐amyloid 1–42 levels than the normoglycemic population.^12^ Some studies have reported a J‐shaped association between HbA1c and dementia incidence.^10^ A meta‐analysis of nine studies found that severe hypoglycemia was associated with an approximately 1.5‐fold risk of incident dementia.^13^

Randomized controlled trials (RCTs) have been inconclusive regarding whether glycemic control is effective for preventing cognitive decline and the development of dementia. However, a recent meta‐analysis of seven RCTs reported that glycemic control delayed cognitive decline, as assessed using the Mini‐Mental State Examination, Wechsler Memory Scale‐R, and Digit Symbol Substitution Test approaches.^14^

### Diabetes and frailty

Diabetes is a disease that predisposes patients to frailty. One meta‐analysis reported that patients with diabetes are 1.48 times more likely to develop frailty than those without.^15^ Conventional risk factors for the development of frailty in patients with diabetes include high HbA1c levels, hypoglycemia or low HbA1c levels, vascular risk factors, macrovascular disease, and abdominal obesity. Patients with diabetes who have HbA1c levels of ≥8.0% are 3.8 times more likely to develop frailty than those with levels of <5.5%.^16^ In a longitudinal study, the risk of incident frailty was 1.30 times higher for an HbA1c level of 8.2%, and 1.41 times higher for one of 6.9%—indicating a U‐shaped association.^17^ In a longitudinal study conducted in Taiwan, the incidence of frailty increased with the frequency of severe hypoglycemia.^18^

In our longitudinal study of older patients with cardiometabolic disease in a frailty clinic, the following new risk factors for incident frailty were identified: low speed of ground reaction force during the sit‐to‐stand movement,^19^ elevated levels of growth differentiation factor 15 (GDF15),^20^, ^21^ and local abnormalities in cerebral white matter integrity.^22^

GDF15 is associated with inflammation, oxidative stress, and insulin resistance and may represent a possible marker of age‐related mitochondrial dysfunction. We found that high GDF15 levels were associated with sarcopenia in patients with diabetes,^20^ and that GDF15 predicted incident frailty in a longitudinal study.^21^

Frailty has been reported to be a risk factor for the development of cardiovascular disease.^23^ This finding suggests that atherosclerosis contributes to the development of frailty. Alterations in the integrity of white matter in the anterior thalamic radiation on diffusion tensor imaging of brain, which reflects early brain microvascular abnormalities, have been found to be associated with incident frailty.^22^ Such white matter lesions are also associated with sarcopenia and markers of atherosclerosis, such as the ankle brachial index, in patients with diabetes.^24^, ^25^ Furthermore, white matter lesions are associated with the glycoalbumin‐to‐HbA1c ratio and a low body mass index (BMI),^25^ suggesting that high glucose variability and malnutrition may influence abnormalities in local white matter fibers in the brain.

Frailty and cognitive impairment have common risk factors, including hyperglycemia, hypoglycemia, vascular risk factors, abdominal obesity, cerebral white matter lesions, decreased physical activity, low nutritional status, and decreased social interaction.^26^ They also have common etiologies, such as insulin resistance, inflammation, oxidative stress, mitochondrial dysfunction, and atherosclerosis. Therefore, patients must take common measures to treat both conditions—such as appropriate glycemic control, management of vascular risk factors, exercise (including resistance exercise), nutritional support, and social participation.

## Diabetes and comorbidity or multimorbidity

In older diabetes, retinopathy, nephropathy, neuropathy, stroke, and cardiovascular disease represent classical diabetes‐specific comorbidities, while heart failure, sarcopenia, sarcopenic obesity, fractures, depression, and periodontal and malignant diseases are considered nonspecific comorbidities.

### Diabetes and sarcopenia or sarcopenic obesity

One meta‐analysis reported that patients with diabetes are 1.55 times more likely to suffer from sarcopenia than those without diabetes.^27^ In a meta‐analysis of risk factors for sarcopenia in patients with diabetes, older age, low BMI, high HbA1c level, and osteoporosis were determined to be predictors of incident sarcopenia, whereas metformin use and protein intake were protective factors.^28^ Patients with diabetes are also more likely to develop sarcopenic obesity.^29^, ^30^ Insulin resistance and inflammation are thought to represent the mechanisms underlying the relationship between sarcopenia and obesity. Compared with simple obesity, sarcopenic obesity is associated with a higher risk of impaired instrumental ADLs, frailty, falls, development of cardiovascular disease, and death.^31^, ^32^ A longitudinal study reported a 2.94‐fold risk of falls or death and a 6.02‐fold risk of cardiovascular disease in older patients with diabetes mellitus and sarcopenic obesity.^29^

### Stroke

Because stroke is associated with several geriatric syndromes—such as cognitive impairment, frailty, falls, depression, and ADL limitations—managing vascular risk factors is important for stroke prevention. Diabetes is likely to cause cerebral white matter lesions, which in turn lead to stroke, cognitive decline, and functional disability.^33^ Therefore, assessing brain white matter is important in older adults with diabetes who have vascular risk factors.

### Congestive heart failure

Older adults with diabetes are also more likely to have a higher risk for the development and progression of heart failure. A 10‐year longitudinal study of 3.25 million individuals reported that patients with diabetes are more likely to be hospitalized for heart failure as they age—particularly those with type 1 diabetes, who are more likely to be hospitalized for heart failure and have a higher 30‐day mortality rate.^34^

### Multimorbidity

**Figure 3:**
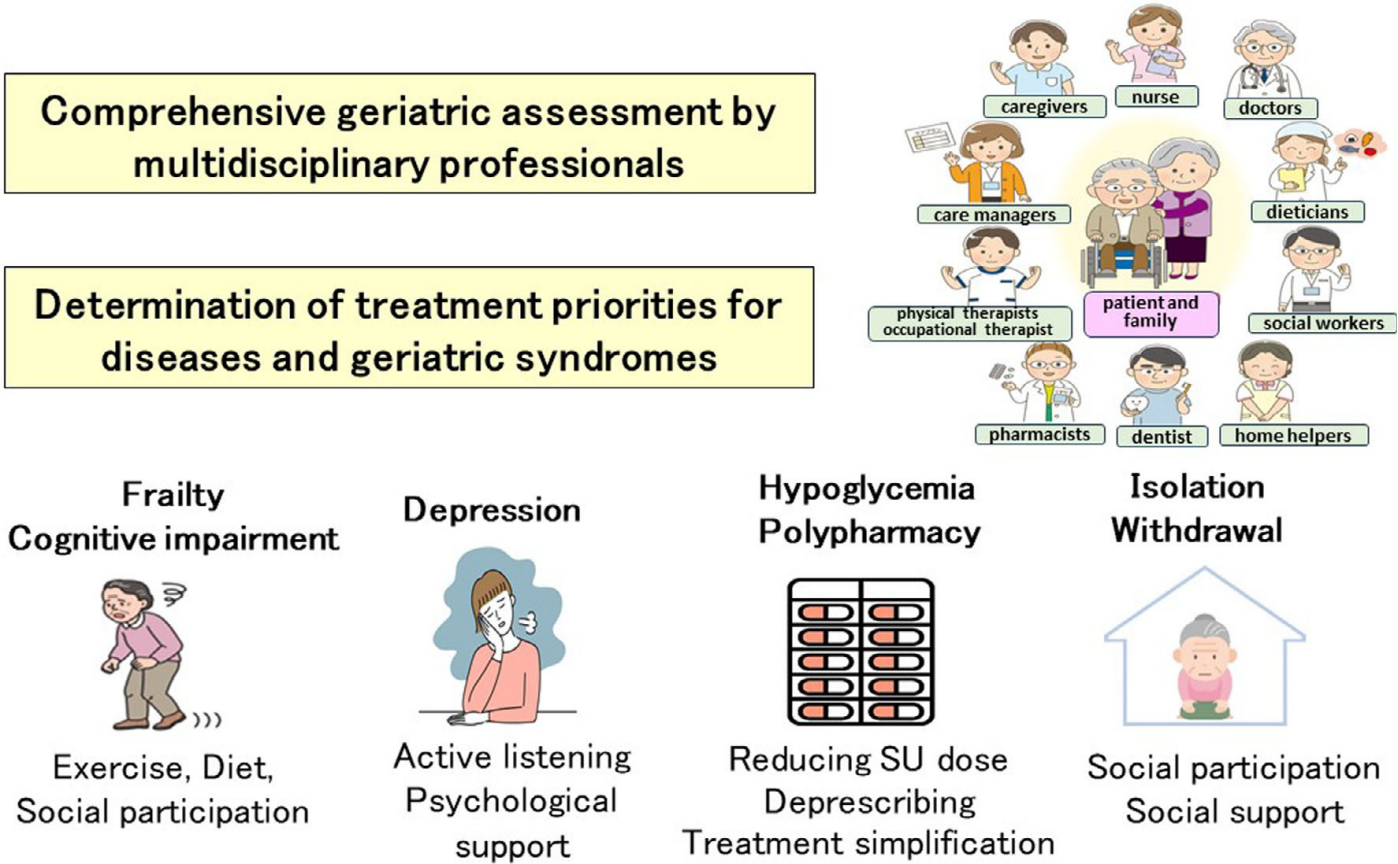
Care of older adults with diabetes and multimorbidity. For older adults with diabetes and multimorbidity, physicians should perform CGA in collaboration with multidisciplinary staff; determine treatment priorities of diseases and geriatric syndromes; and take measures to prevent frailty, cognitive impairment, depression, hypoglycemia, polypharmacy, isolation, and withdrawal.

Multimorbidity is defined by the World Health Organization (WHO) as the coexistence of ≥2 long‐term conditions (i.e., chronic conditions).^35^ Recently, the term chronic conditions has been used to describe diseases and geriatric syndromes such as functional disabilities, sensory impairment, and frailty. Diabetes represents a common cause of multimorbidity. In data from 18 968 older patients with diabetes, the median number of comorbidities was five.^36^ In the Japan Diabetes comPRehensive database project based on an Advanced electronic Medical record System (J‐DREAMS) study of 10 151 patients with diabetes, 53.5% of those aged ≥75 years had ≥4 comorbidities compared with 34.7% of those aged <65 years.^37^ In another meta‐analysis, multimorbidity in diabetes was associated with an increased risk of death in 15 of 17 studies, and with hypoglycemia in 9 of 10 studies.^38^ A 5‐year longitudinal study of patients with type 2 diabetes in the UK Biobank registry found that, as the number of comorbidities increased, not only the risk of death, but also the risks of hypoglycemia, falls or fractures, and major cardiovascular events increased.^4^ The Japanese Elderly Intervention Trial (J‐EDIT) study also found an increased risk of death in patients with multimorbidities and ≥4 of the following eight comorbidities: retinopathy, nephropathy, neuropathy, cerebrovascular disease, ischemic heart disease, liver disease, malignant disease, and depression.^39^ Multimorbidity is associated with physical impairment, frailty, cognitive impairment, depression, polypharmacy, hypoglycemia, and the need for social services. Although evidence for the treatment of multimorbidity is lacking, some studies have shown that Comprehensive Geriatric Assessment (CGA)‐based care and tailored interventions by multidisciplinary teams can delay the progression of frailty in community‐dwelling older adults with multimorbidity.^40^ For older adults with diabetes and multimorbidity, physicians should perform the CGA in collaboration with multidisciplinary staff; determine treatment priorities for diseases and geriatric syndromes; and take measures to prevent frailty, cognitive impairment, depression, hypoglycemia, polypharmacy, isolation, and withdrawal (Fig. 3).

### Frailty and multimorbidity

“Frailty in the deficit accumulation model,” which is different from phenotype frailty, is based on the accumulation of deficits such as chronic diseases and geriatric syndromes.^41^ The concept of “Frailty in the deficit accumulation model” is similar to that of multimorbidity, wherein deficits are replaced by chronic conditions. The frailty index (FI), an index of the deficit accumulation model, is based on the percentage of item with deficits out of 50‐70 items. Based on the percentage of items that correspond to the number of deficits in 50–70 items. One meta‐analysis reported that a higher FI with multimorbidity was significantly associated with increased risks of hypoglycemia, falls or fractures, cardiovascular disease, and mortality.^42^ A multifactorial intervention in patients with obesity and type 2 diabetes in the Look Ahead study improved the FI after 8 years.^43^ Regarding the management of patients with diabetes with severe “Frailty in the deficit accumulation model,” prioritizing the treatment of diseases and geriatric syndromes, and having CGA assessments conducted by multidisciplinary staff, using a similar approach to that for those with multimorbidity, is important.

### Dietary management

**Figure 4:**
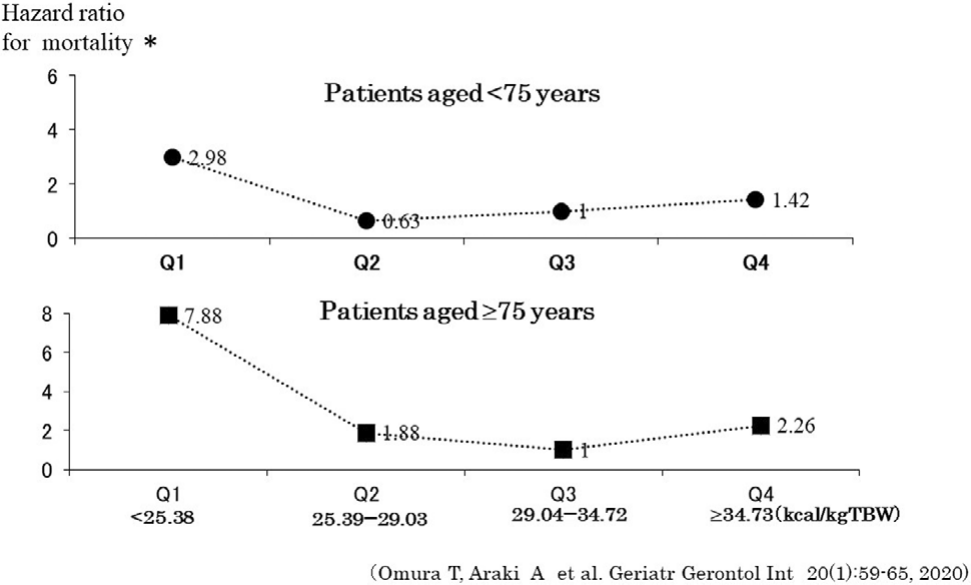
Energy intake and mortality in older adults with diabetes by age group at 75 years of age. *Adjusted for age, sex, BMI, HbA1c, SBP, LDL‐C, eGFR, history of IHD, history of stroke, history of hypoglycemia, and protein intake per body weight. BMI, body mass index; IHD, ischemic heart disease; LDL‐C, low density lipoprotein cholesterol; SBP, systolic blood pressure. Mortality was lowest in the ~29–35 kcal/kg group in those aged ≥75 years, and in the 25–29 kcal/kg group in those aged <75 years, suggesting that increased energy intake is desirable in adults with diabetes aged ≥75 years.

Older adults with diabetes are prone to malnutrition—which may lead to frailty, cognitive dysfunction, decreased ADLs, and an increased risk of mortality.^44^, ^45^ Considering the prevention of frailty and sarcopenia, dietary therapies for diabetes should include adequate energy and sufficient protein and vitamin intake to prevent malnutrition. Total energy intake (kcal/day) can be obtained by multiplying the target body weight (TBW; kg), obtained using the formula [height (m)]^2^ × 22~25. In addition to blood glucose control and the prevention of atherosclerotic diseases, exercise therapy in older adults is thought to be effective for preventing frailty, sarcopenia, and dementia, as well as for improving quality of life. By an energy coefficient (kcal/kg) of 25–35, depending on the patient's amount of physical activity.^46^ Both extreme energy restriction and overeating should be noted in older adults with diabetes. In the J‐EDIT study on older patients with diabetes, the risk of death increased both in the group with an energy intake per TBW of ≤25 kcal/kg and in the group with >35 kcal/kg.^47^ A stratified analysis reported that mortality was lowest in the ~29–35 kcal/kg group in those aged ≥75 years, and in the 25–29 kcal/kg group in those aged <75 years, suggesting that increased energy intake is desirable in adults with diabetes aged ≥75 years (Fig. 4).^47^

A protein intake of at least 1.0 g/kg body weight is recommended to maintain muscle mass and function in older adults, and more protein should be consumed in cases of undernutrition, severe disease, and in those undergoing resistance exercise.^48^ In a longitudinal study of older women with diabetes, those who consumed >1.0 g/kg body weight of protein had less knee extension and physical function loss than those who consumed <1.0 g/kg.^49^ Because leucine stimulates muscle protein synthesis, leucine‐rich foods such as meat, fish, dairy products, eggs, and soy products should be recommended.

Although there is concern that protein intake may worsen renal function, evidence regarding the effects of protein restriction in older adults is lacking. The lowest quartile (<0.92 g/kg body weight) had the highest risk of death, and the group with 1.15–1.41 g/kg body weight had the lowest risk of death; however, no association was found between protein intake and impaired renal function.^50^ Therefore, in the absence of severe renal dysfunction, older patients aged >75 years must consume adequate levels of protein. In cases of severe renal impairment, protein restriction should be considered; however, if there is a risk of sarcopenia, protein intake to maintain muscle mass may be appropriate. The intake of a variety of foods may reduce the risk of frailty through energy and protein intake. The variety of an individual's food intake can be assessed using the Dietary Variety Score (DVS), which is based on whether 10 particular food items are consumed almost every day.^51^ In a cross‐sectional study of community‐dwelling residents, the prevalence of Japanese version of Cardiovascular Health Study (J‐CHS) frailty in the group with diabetes and low DVS (≤4 points) was ~5 times higher than in the control group without diabetes and high DVS, and lower dietary variety was associated with frailty.^52^

## Exercise therapy

In addition to blood glucose control and the prevention of atherosclerotic diseases, exercise therapy in older adults is thought to be effective for preventing frailty, sarcopenia, and dementia, as well as for improving quality of life. In terms of aerobic exercise, patients should be instructed to aim for 1 h/day, at least 4–5 days per week. Resistance training and multi‐component exercises are recommended to prevent frailty and sarcopenia. Resistance exercises should be conducted at least twice per week using community exercise classes or rehabilitation services provided by long‐term care insurance. Multicomponent exercises begin with flexibility exercises, followed by a combination of resistance, balance, and aerobic exercises of increasing intensities. Multicomponent exercise not only improves physical performance—including in the Short Physical Performance Battery (SPPB), a popular measure of frailty—but also improves cognitive function after 24 months of exercise, in older patients with diabetes and impaired lower limb function.^53^

In addition, multifactorial interventions including exercise, diet, and diabetes treatment are effective in preventing frailty. An RCT of patients with type 2 diabetes aged ≥70 years reported that multifactorial interventions, including diet, exercise, and optimal control of glucose and blood pressure, significantly improved the SPPB score after 12 months.^54^

## Therapy with hypoglycemic drugs

When administering hypoglycemic drug therapy, it is important to (i) reduce the risk of adverse events such as hypoglycemia, (ii) select drugs and adjust doses based on the evaluation of renal function, (iii) consider the risks and benefits of sodium glucose cotransporter‐2 (SGLT2) inhibitors and glucagon‐like peptide‐1 (GLP‐1) receptor agonists for cardiovascular disease, and (iv) simplify the treatment in patients with poor adherence.

### Prevention of hypoglycemia

Older patients with diabetes are more prone to severe hypoglycemia—particularly those aged >80 years.^55^ One meta‐analysis reported that severe hypoglycemia is a risk factor for microvascular and macrovascular complications, dementia, falls, fractures, and cardiovascular or all‐cause mortality.^13^

Risk factors for severe hypoglycemia include (i) geriatric syndromes, (ii) comorbidities and multimorbidity, (iii) complex glucose dynamics, and (iv) socioeconomic status (Fig. 2 **)**. Severe hypoglycemia is more likely to occur in patients with geriatric syndromes such as cognitive impairment or dementia,^56^, ^57^, ^58^ ADL impairment,^58^ and depression,^59^ leading to a bidirectional relationship and a vicious cycle. In older veterans with diabetes, severe hypoglycemia is more likely to occur as the severity of cognitive impairment increases.^57^ In one longitudinal study, ADL impairment, cognitive impairment, and glycemic variability (1.5 AG) were identified as risk factors for severe hypoglycemia.^58^ Low BMI^60^ and polypharmacy (i.e., taking >5 prescription drugs regularly)^61^ have been associated with hypoglycemic events.

Those with chronic kidney disease (CKD),^62^ coronary artery disease,^63^ heart failure,^64^ or stroke/transient ischemic attack^64^ are more likely to develop hypoglycemia. An increased number of chronic conditions in cases of multimorbidity represents a predictor of hypoglycemic events.^4^

Increased glycemic variability predisposes patients to severe hypoglycemia. Patients with type 1 diabetes and a history of severe hypoglycemia have hypoglycemia unawareness and greater glycemic variability at continuous glucose monitor (CGM).^5^ In older adults with type 1 diabetes, the use of blood glucose variability (CV) and the glucose management indicator (GMI) in CGM can better identify individuals at a higher risk of hypoglycemia (<70 mg/dL or <54 mg/dL) compared with HbA1C alone.^65^

To prevent hypoglycemia in older patients with diabetes, the following protective factors against hypoglycemia should be strengthened. (i) There should be a preferential use of drugs with a low risk of hypoglycemia. (ii) Sulfonylureas (SUs) should be used at the lowest possible dose in combination with other hypoglycemic agents. The lower the renal function, the more SU drugs accumulate, so caution against hypoglycemia is warranted; SU drugs should not be used in patients with estimated glomerular filtration rate (eGFR) values of <30 mL/min/1.73 m^2^. One meta‐analysis reported that gliclazide was associated with approximately one‐ninth as many cases of severe hypoglycemia as glimepiride.^66^ If glimepiride is given at 1–2 mg/day and a dipeptidyl peptidase‐4 (DPP‐4) inhibitor or metformin is added, the glimepiride dose should be reduced by half and eventually to the lowest dose. When metformin was added to SU drugs, the hypoglycemic risks of gliclazide and glimepiride were reduced by 60% and 70%, respectively, compared with that of glibenclamide.^67^ Our practice prefers to prescribe gliclazide at very low doses of 10–20 mg/dL. (iii) Insulin therapy should be simplified and used in combination with other drugs, or GLP‐1 receptor agonists, at lower doses. (iv) Patients and their caregivers should be educated on how to deal with hypoglycemia and sick days. (v) Self‐monitoring blood glucose (SMBG) and CGM should be used to monitor hypoglycemia. A study of CGM in patients with diabetes (median age, 82 years) found at least one episode of hypoglycemia (<70 mg/dL) during a 5‐day period in 33% of insulin‐treated patients and in 44% of patients on other hypoglycemic drugs.^68^ Sharing CGM glucose values between patients and caregivers may be effective for preventing severe hypoglycemia in older patients with diabetes.^69^

### Treatment based on renal function assessment

**Figure 5:**
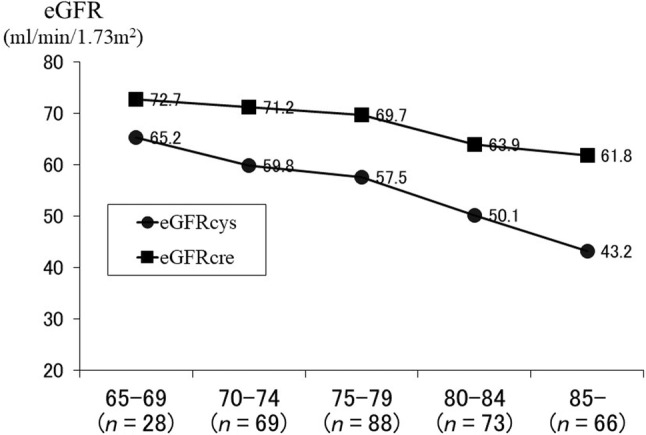
Assessment of renal function using eGFRcre and eGFRcys by age. Renal function was assessed using eGFRcre and eGFRcys in a total of 324 older patients with diabetes mellitus after excluding those with serum creatinine ≥1.5 mg/dL. The difference between eGFRcre and eGFRcys was highest in those aged ≥80 years (*p* < 0.001). eGFRcre (mL/min/1.73 m^2^) = 194 × Cr ^−1.094^ × age (years)^(−0.287)^ (men). eGFRcre (mL/min/1.73 m^2^) = 194 × Cr^−1.094^ × age (years)^(−0.287)^ × 0.739 (women). Cr: serum creatinine (mg/dL). eGFRcys (mL/min/1.73 m^2^) = 104 × CysC^−1.019^ × 0.996 ^age(year)^ − 8 (men). eGFRcys(mL/min/1.73 m^2^) = 104 × CysC^−1.019^ × 0.996^−age(year)^ × 0.929 − 8 (women). CysC: serum cystatin C (mg/L).

When metformin, imeglimin, SGLT2 inhibitors, or SU drugs are used, renal function shoud be assessed using the eGFR to determine if those drugs are indicated and to make dose adjustments. The eGFRcre, based on serum creatinine levels, may be overestimated in older patients with a low BMI or level of skeletal muscle. In a cross‐sectional study of patients with diabetes, the discrepancy between eGFRcre and eGFRcys, based on serum cystatin C, increased with age >80 years (Fig. 5).^70^ The use of eGFRcys has been used to reclassify some patients with diabetes from metformin‐eligible stages to stages 3b and 4 CKD.^71^ Therefore, checking renal function at least once in patients aged >80 years using eGFRcys is important. Metformin use should be discontinued if the eGFR is found to be <30 mL/min/1.73 m^2^, because of the increased risk of lactic acidosis.^72^ If the eGFR is >30 mL/min/1.73 m^2^, 500 mg/day should be used first, before increasing to 1500 mg/day if the eGFR is >60 mL/min/1.73 m^2^. If the eGFR is <45 mL/min/1.73 m^2^, the dosage should be reduced. Therefore, metformin and imeglimin should be used with caution for gastrointestinal symptoms.

### Treatment simplification

Simplifying treatment as a countermeasure against decreased adherence because of cognitive impairment is important. For oral hypoglycemic agents, reducing the complexity of drug prescriptions is important and can be done by (i) reducing the number and frequency of doses, (ii) consolidating the timing of dosing, (iii) using single packets (excluding SU drugs), and (iv) using combination drugs. Insulin therapy can be simplified by reducing the number of insulin units and switching to (i) once‐daily basal insulin analogues, (ii) once‐weekly GLP‐1 receptor agonists, or (iii) a combination of basal insulin and GLP‐1 receptor agonists. In a single‐arm intervention study of older patients with type 2 diabetes with ≥2 insulin injections, hypoglycemia in CGM (<70 mg/dL) decreased from 277 to 97 min after 8 months of insulin treatment simplification involving switching from multiple insulin injections to once‐daily glargine (a once‐daily insulin dose combined with oral hypoglycemic drugs).^73^ In an RCT study of three groups of insulin‐treated patients with diabetes, namely (i) continued intensified therapy, (ii) the addition of an SGLT2 inhibitor to a once‐daily basal insulin analog, and (iii) changes to the combination of basal insulin and GLP‐1 receptor agonists, glycemic control was similar, but the SGLT2 inhibitor and combination groups had their number of insulin units and hypoglycemia levels decreased by >50%.^74^ The key to simplifying insulin therapy is to use basal insulin in combination with metformin, DPP‐4 inhibitors, SGLT2 inhibitors, and GLP‐1 receptor agonists—as well as to reduce the insulin dose when blood glucose is <80 mg/dL on ≥2 occasions in 1 month. Once‐weekly insulin could be an effective way to simplify treatment even for older patients. Once‐weekly insulin icodec or basal insulin Fc (insulin efsitora α) has been reported to provide better or similar glycemic control compared with a once‐daily basal insulin analog, and the rate of hypoglycemia is similar or lower.^75^, ^76^

### Additional benefits and risks of SGLT2 inhibitors and GLP‐1 receptor agonists

The use of SGLT2 inhibitors and GLP‐1 receptor agonists has additional benefits in terms of preventing cardiovascular disease and composite renal events in older adults. The use of SGLT2 inhibitors reduces the risk of heart failure. In one meta‐analysis, the use of SGLT2 inhibitors in older adults with diabetes reduced the risk of three‐point major adverse cardiovascular events (MACE), stroke, heart failure, and composite renal endpoints by 13, 17, 38, and 43%, respectively.^77^ In propensity‐matched cohorts, SGLT2 inhibitor use was associated with a lower risk of 1‐year mortality and heart failure readmission than DPP‐4 inhibitor use, even in patients with diabetes aged >75 years.^78^ The use of GLP‐1 receptor agonists in patients aged ≥65 years also reduces the risk of MACE, cardiovascular death, and stroke by 14, 19, and 18%, respectively (similarly to the case in patients aged <65 years).^77^ These trends have also been seen in patients aged ≥75 years. Notably, in a propensity score‐matched study of SGLT2 and DPP‐4 inhibitor users, the risk reduction in terms of major cardiovascular outcomes with SGLT2 inhibitor use among those with frailty (FI >0.25) was greater than that among those without frailty.^79^ A recent meta‐analysis reported that use of SGLT2 inhibitors and GLP‐1 receptor agonists reduced the risk of developing dementia by 38 and 28%, respectively.^80^ Further investigations of this topic through RCT studies are warranted.

SGLT2 inhibitors should be used, even in older patients at risk of cardiovascular disease, heart failure, or CKD. However, these should be administered with caution to older patients aged >75 years, or to those with geriatric syndromes. Patients using SGLT2 inhibitors should be carefully monitored for dehydration, genital tract infections, and ketoacidosis. Malnutrition (i.e., unintentional weight loss and low BMI) should preclude the use of SGLT2 inhibitors. Exercise—particularly resistance exercise—is recommended to prevent sarcopenia when using weight‐reducing agents.

### Type 1 diabetes in older adults

Notably, new cases of type 1 diabetes can occur in older adults. According to the National Database Survey of Japan (2014–2017), the number of cases of type 1 diabetes per 100 000 people in the age group of 40–59 years ranged between 4.99 and 5.70, whereas the number of cases in the age group of ≥60 years ranged between 3.31 and 3.48, exhibiting a slight downward trend.^81^ The development of acute‐onset or fulminant type 1 diabetes associated with the use of immune checkpoint inhibitors for malignant diseases should also be considered, even in older adults.^82^

The use of CGM in older patients with type 1 diabetes in Japan is only 24.3%.^83^ However, its use in older adults with type 1 diabetes may reduce hypoglycemia. In One study treated two groups of patients with type 1 diabetes ≥60 years for 26 weeks in a real‐time CGM group and a control group using the SMBG and compared the time <70 mg/dL (TBR) and time between 70 and 180 mg/dL (TIR). The TBR in the CGM group decreased from 5.0% to 2.6% and TIR increased from 56% to 64%—resulting in less hypoglycemia and better blood glucose control compared with the control group.^84^ One meta‐analysis of 165 older patients with type 1 diabetes (mean age, 70 years) also reported that the insulin pump and CGM groups had less unintentional hypoglycemia and significantly lower HbA1c levels, time of low glucose (< 54 mg/dL), and CV compared with the multiple daily injection and SMBG groups.^85^ For insulin pump therapy with CGM to be widely adopted in older patients with type 1 diabetes, it is necessary to develop a simpler insulin pump and to secure human resources and financial support for its management.

## Goals of glycemic control in older adults with diabetes

**Figure 6:**
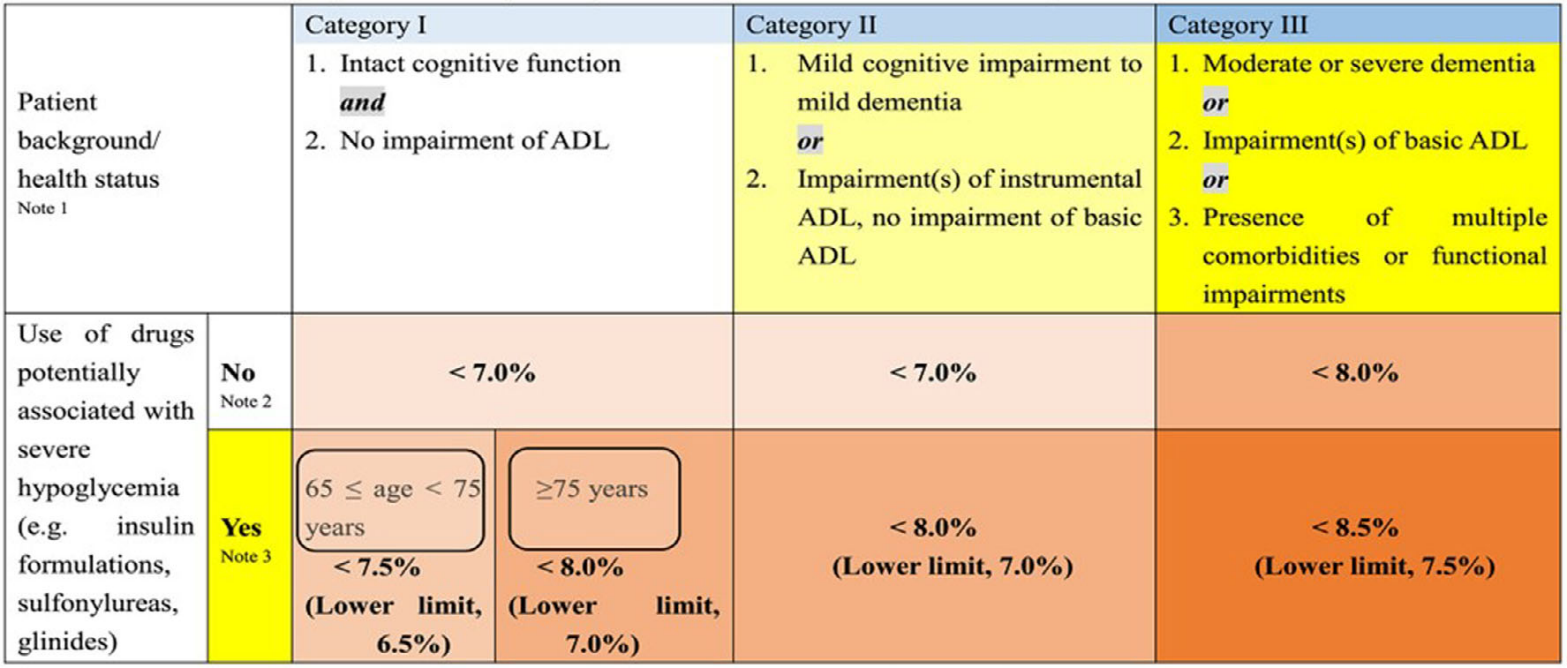
Glycemic targets for elderly patients with diabetes set by the Japan Diabetes Society (JDS)/Japan Geriatrics Society (JGS). The glycemic target is to be determined for each patient by taking into account his/her age, duration of diabetes, risk for hypoglycemia, and any support available to the patient, as well as the patient's cognitive function, basic/instrumental ADL, and comorbidities/functional impairments, if elderly, while noting that the potential risk of hypoglycemia increases with age in each patient. ADL, activities of daily living. Note 1 Refer to the Japan Geriatrics Society website^[^ ^10^, ^11^ ^]^, for the evaluation of cognitive function, basic ADL (e.g., self‐care abilities such as dressing, transferring, bathing, and toileting), and instrumental ADL (e.g., abilities to maintain an independent household such as shopping, meal preparation, taking medication, and handling finances). In end‐of‐life care, priority is to be given to preventing significant hyperglycemia and subsequent dehydration and acute complications through appropriate therapeutic measures. Note 2 As in other age groups, the glycemic target is set at <7.0% in the elderly for preventing diabetic complications. However, this could be set at <6.0% for those thought likely to achieve glycemic control through diet and exercise therapy alone or for those likely to achieve glycemic control with drug therapy without adverse reactions, or at 8.0% for those in whom intensifying therapy may prove difficult. In either case, no lower limit is specified for the glycemic target. A glycemic target of <8.5% may be allowed for patients thought to be in Category III and therefore at risk of developing adverse reactions to multi‐drug combination therapy or for those with serious comorbidities or poor social support. Note 3 In patients in whom priority should be given to preventing the onset/progression of diabetic complications owing to their duration of disease, the glycemic target or its lower limit may be set for each elderly patient with appropriate measures in place to prevent severe hypoglycemia. Current treatments are to be continued for those younger than 65 years of age despite their HbA1c values falling below their glycemic target or lower limit while on therapy, but care needs to be taken to monitor these patients for potential severe hypoglycemia. Glinides may be classified as drugs unlikely to be associated with severe hypoglycemia, as the onset of severe hypoglycemia varies depending on the type and amount of glinide used in a particular patient relative to the patient's glucose level.

The glycemic control goals for diabetes in older adults set by the Japan Geriatrics Society (JGS) and the Japan Diabetes Society (JDS) are classified into three categories based on the evaluation of cognitive function, ADL, and comorbidities. They are also set according to age and the use of drugs (e.g., SU drugs, insulin, and others) that may pose a risk of severe hypoglycemia (Fig. 6).^86^ If a drug that poses a risk of severe hypoglycemia is used, the target value should be slightly lenient, and a lower target limit should be set. For example, the targets for Category I and Category II in much older patients aged ≥75 years are HbA1c <8.0%, and the lower limit is 7.0%. On the other hand, if the risk of severe hypoglycemia is low, the goals for Categories I and II are HbA1c <7.0%, with no lower limit.

Glycemic control goals for older adults in the JDS/JGS,^86^ the Endocrine Society,^87^ and the American Diabetes Association (ADA),^88^ are similarly based on the three categories using cognitive function, ADLs, comorbidities, and so on, but similarly based on the three categories of cognitive function, ADLs, but there are some notable differences. HbA1c targets in the US Veterans Affairs and Department of Defense (VA/DoD) guidelines have lower limits and are determined by diabetic complications and life expectancy based on comorbidities, but not by ADLs and cognitive function.^89^

In the Endocrine Society Clinical Practice Guideline from the European Society of Endocrinology, the Gerontological Society of America, and the Obesity Society,^87^ glucose targets and lower limits were determined by three categories similar to those in the JDS/JGS guideline. The categories were based on ADL impairment, cognition, the number of non‐diabetic chronic conditions, residence in a long‐term care facility, and the presence of end‐stage disease.^87^ In the ADA, Category III does not set a specific HbA1c target and avoids prominent hyperglycemia and hypoglycemia^87^—whereas in Japan, the target HbA1c is 8.5%, and the lower limit is 7.5% for Category III individuals at risk of hypoglycemia.^86^ According to the JDS/JGS, the end of life is considered separate from Category III, and it should be recommended to treat so that significant hyperglycemia and hypoglycemia are avoided. In Japan, institutionalization is not used for categorization. But the categorization is evaluated by ADL impairment, cognition. One study reported that the glycemic control of patients with type 2 diabetes in 72 nursing homes in Japan was stringent, with 76.8% having HbA1c levels of <*7.0%*.^90^ In that study, HbA1c < 7% was associated with a higher severity of dementia, particularly in those patients who were using insulin.^90^ Therefore, glycemic control in patients with diabetes who reside in long‐term care facilities should be optimized with reference to lower target limits.

In the J‐EDIT study of older patients with diabetes, the mortality risk increased with advancing categories, with the risk of mortality in Category II and III patients being 1.8 and 3.1 times higher, respectively, compared with Category I.^39^ These results suggest that setting more flexible glycemic control targets as a patient's category classification progresses is appropriate. In a study of memory clinic patients, HbA1c levels above and below the JDS/JGS guideline target range were associated with ADL decline and mortality.^91^, ^92^ The increase (>60%) in time above or below the VA/DoD guideline target range was associated with the incidence of macrovascular complications and mortality, although the hazard ratios were only 1.02–1.12.^93^ However, in a retrospective longitudinal study of 63 429 older adults with diabetes, an association between HbA1c levels above the Endocrine Society target range and the risk of diabetic complications, including hypoglycemia and mortality, was observed only in the good and immediate health categories, but not in the poor health category.^94^ HbA1c levels below the target range were associated with complications only in the good health category. Further research is needed on setting glycemic target ranges, including upper and lower limits by category, based on evidence of diabetic complications, functional prognosis, and mortality.

The Dementia Assessment Sheet for Community‐based Integrated Care System‐8 items (DASC‐8) can be used to perform this categorization.^95^ The DASC‐8 consists of eight questions on memory, time perception, instrumental ADLs (shopping, transportation, and money management), and basic ADLs (toileting, eating, and mobility) on a four‐point scale (Table 1).

**Table 1:** The Dementia Assessment Sheet for Community‐based Integrated Care System‐8 items (DASC‐8)

		1 point	2 points	3 points	4 points	Topic
A	Do you have the impression that he/she forgets a lot of things?	a. No	b. Yes, a little	c. Yes	d. Yes, a lot	Introductory questions (no points)
B	Compared to last year, do you have the impression that he/she forgets more things?	a. No	b. Yes, a little	c. Yes	d. All the time	
1	Does he/she forget where he/she puts things such as his/her wallet or keys?	a. Never	b. Sometimes	c. Frequently	d. Always	Memory
2	Does he/she forget what day and month it is?	a. Never	b. Sometimes	c. Frequently	d. Always	Orientation
3	Can he/she buy things by himself/herself?	a. Yes, without difficulty	b. Can most of the time	c. Can't most of the time	d. Not at all	IADL outside the home
4	Can he/she use the bus, the train or a car by himself/ herself to go out?	a. Yes, without difficulty	b. Can most of the time	c. Can't most of the time	d. Not at all	
5	Can he/she pay the rent and bills, withdraw money or make a deposit by himself/ herself?	a. Yes, without difficulty	b. Can most of the time	c. Can't most of the time	d. Not at all	
6	Can he/she use the toilet by himself/herself?	a. Yes, without difficulty	b. Needs supervision or instructions	c. Needs partial assistance	c. Needs full assistance	BADL
7	Can he/she eat on his/her own?	a. Yes, without difficulty	b. Needs supervision or instructions	c. Needs partial assistance	c. Needs full assistance	
8	Can he/she move around the house by himself/herself?	a. Yes, without difficulty	b. Needs supervision or instructions	c. Needs partial assistance	c. Needs full assistance	

*Note*: Total score _/32 points. Based on the total score of DASC‐8, individuals can be classified into the three categories for determining the glycemic targets for elderly patients with diabetes: Japan Diabetes Society (JDS)/Japan Geriatrics Society (JGS) Joint Committee on Improving Care for Elderly Patients with Diabetes.^95^ (Geriatr Gerontol Int 2016; 16: 1243–1245), Diabetol Int 2016; 7(4): 331–333. Category I; DASC‐8 total score of 10 or less; Category II, DASC‐8 total score of 11–16; Category III, DASC‐8 total score of 17 or more. Because the DASC‐8 is a simple screening test for cognitive and daily function, it is desirable that the assigned category should be confirmed by the detailed examination of cognition, IADL, and BADL.

Abbreviations: BADL, basic activities of daily living; IADL, instrumental activities of daily living.

## Individualized treatment based on categorization and CGA

The prevalence of frailty, dementia, depression, malnutrition, and poor medication adherence increases with the advancement of the categorization stage, according to the DASC‐8.^96^ Our previous cross‐sectional study found that Category II patients had a higher prevalence of frailty;^96^ however, one longitudinal study also found that Category II patients without frailty were at a higher risk of frailty and certified long‐term care needs, or mortality.^97^ It is important to take the following measures: prevention of frailty and cognitive impairment, psychological intervention, nutritional support, treatment simplification, and social participation or support from the Category II stage.

**Figure 7:**
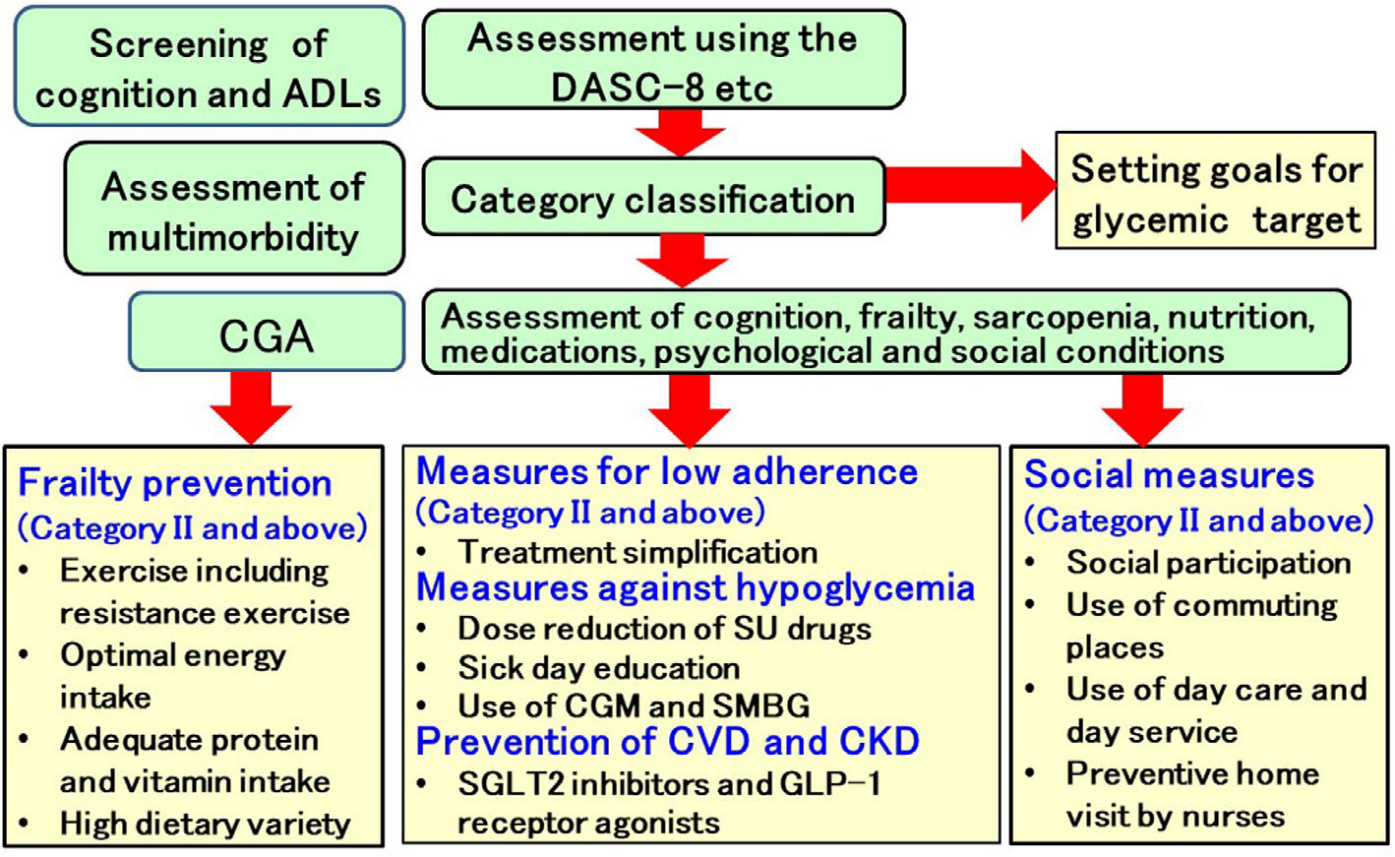
Individualized treatment for older adults with diabetes. Categorization using the DASC‐8 can be used to set glycemic control goals and determine treatment strategies for older patients with diabetes when combined with the CGA, which includes assessment of cognition, frailty, psychology, nutrition, medications, social status, and multimorbidity. Based on the CGA, frailty prevention, treatment simplification, and social participation or services should be implemented in patients in Category II and above. Measures against hypoglycemia and prevention of cardiovascular disease and CKD should also be promoted. Treatment based on categorization and CGA by multidisciplinary professionals would be an individualized treatment for older adults with diabetes. CGA, Comprehensive Geriatric Assessment; CKD, chronic kidney disease; CVD, cardiovascular disease; DASC‐8, Dementia Assessment Sheet for Community‐based Integrated Care System‐8 items.

Categorization can be used to set glycemic control goals and determine treatment strategies for older patients with diabetes by combining it with the CGA—which includes an assessment of cognition, frailty, psychology, nutrition, medications, social status, and multimorbidity (Fig. 7). One meta‐analysis found that CGA increased the probability of staying at home after discharge in hospitalized patients aged >65 years.^98^ Although little evidence is available for CGA in older adults with diabetes, the intervention group—in which patients were assessed for barriers to treatment (e.g., diet, physical activity, cognitive function, depression, hypoglycemia, medications, and social problems) and were educated on how to address them—showed improved HbA1c levels, diabetes‐related distress, 6‐min walking time, and self‐care behaviors.^99^

Based on the CGA, diet and exercise therapy as measures against frailty should be implemented at the Category II stage (Fig. 7). In addition, measures to simplify treatment of oral hypoglycemic agents and insulin for decreased adherence should be implemented. Countermeasures against side effects such as hypoglycemia are also important in the Category II stage. Additionally, social participation should be encouraged in patients of Category II and above, and social services such as preventive home visits by nurses should be introduced as needed. The therapy based on the categorization and CGA by multidisciplinary professionals would be an individualized treatment for the older adults with diabetes mellitus.

## Disclosure statement

AA has received speaker honoraria from Sumitomo Pharma Co. Ltd, Ono Pharmaceutical Co. Ltd, and Novo Nordisk Pharma Ltd.

## Author contributions

AA: study design, data analysis, interpretation, and acquisition, and manuscript preparation.

## Ethics statement

This study was conducted in accordance with the Declaration of Helsinki, and the protocol was approved by the Ethics Committee of the Tokyo Metropolitan Institute for Geriatrics and Gerontology (no. R15‐20).

## Funding

This study was supported by a grant from Research Funding for Longevity Science (28‐30) from the National Center for Geriatrics and Gerontology in Japan.

## Data Availability

Data sharing is not applicable to this article as no new data were created or analyzed in this study.

## References

[1] Magliano DJ , Boyko EJ . IDF Diabetes Atlas 10th edition scientific committee. IDF DIABETES ATLAS [Internet]. 10th edn. Brussels: International Diabetes Federation; 2021. Available from: https://www.ncbi.nlm.nih.gov/books/NBK581934/

[2] The Ministry of Health, Labour, and Welfare . The National Health and Nutrition Examination Survey 2019, Available at: https://www.mhlw.go.jp/content/10900000/000687163.pdf [Cited 6 Jul 2004.] (in Japanese).

[3] Araki A , Ito H . Diabetes and geriatric syndromes. Geriatr Gerontol Int 2009; 9: 105–114. 19740352 10.1111/j.1447-0594.2008.00495.x

[4] Hanlon P , Jani BD , Butterly E *et al*. An analysis of frailty and multimorbidity in 20,566 UK biobank participants with type 2 diabetes. Commun Med (Lond) 2021; 1: 28. 35602215 10.1038/s43856-021-00029-9 PMC9053176

[5] Weinstock RS, DuBose SN, Bergenstal RM , et al. Risk Factors Associated With Severe Hypoglycemia in Older Adults With Type 1 Diabetes. Diabetes Care. 2016; 39: 603–610. 26681721 10.2337/dc15-1426

[6] Zinman B , Marso SP , Poulter NR *et al*. Day‐to‐day fasting glycaemic variability in DEVOTE: associations with severe hypoglycaemia and cardiovascular outcomes (DEVOTE 2). Diabetologia 2018; 61: 48–57. 28913575 10.1007/s00125-017-4423-z PMC6002963

[7] Karter AJ , Parker MM , Huang ES *et al*. Food insecurity and hypoglycemia among older patients with type 2 diabetes treated with insulin or sulfonylureas: the Diabetes & Aging Study. J Gen Intern Med 2024. 10.1007/s11606-024-08801-y. PMC11436613 38767746

[8] Zhang X , Norris SL , Gregg EW , Beckles G . Social support and mortality among older persons with diabetes. Diabetes Educ 2007; 33: 273–281. 17426302 10.1177/0145721707299265

[9] Xue M , Xu W , Ou YN *et al*. Diabetes mellitus and risks of cognitive impairment and dementia: a systematic review and meta‐analysis of 144 prospective studies. Ageing Res Rev 2019; 55: 100944. 31430566 10.1016/j.arr.2019.100944

[10] Rawlings AM , Sharrett AR , Albert MS *et al*. The Association of Late‐Life Diabetes Status and Hyperglycemia with Incident Mild Cognitive Impairment and dementia: the ARIC study. Diabetes Care 2019; 42: 1248–1254. 31221696 10.2337/dc19-0120 PMC6609963

[11] Li FR , Yang HL , Zhou R *et al*. Influence of diabetes duration and glycemic control on dementia: a cohort study. J Gerontol A Biol Sci Med Sci 2021; 76: 2062–2070. 34331763 10.1093/gerona/glab221

[12] McIntosh EC , Nation DA . Alzheimer's disease neuroimaging initiative. Importance of treatment status in links between type 2 diabetes and Alzheimer's disease. Diabetes Care 2019; 42: 972–979. 30833374 10.2337/dc18-1399 PMC6489115

[13] Mattishent K , Loke YK . Meta‐analysis: association between hypoglycemia and serious adverse events in older patients treated with glucose‐lowering agents. Front Endocrinol (Lausanne) 2021; 12: 571568. 33763024 10.3389/fendo.2021.571568 PMC7982741

[14] Lin Y , Gong Z , Ma C , Wang Z , Wang K . Relationship between glycemic control and cognitive impairment: a systematic review and meta‐analysis. Front Aging Neurosci 2023; 15: 1126183. 36776436 10.3389/fnagi.2023.1126183 PMC9909073

[15] Hanlon P , Fauré I , Corcoran N *et al*. Frailty measurement, prevalence, incidence, and clinical implications in people with diabetes: a systematic review and study‐level meta‐analysis. Lancet Healthy Longev 2020; 1: e106–e116. 33313578 10.1016/S2666-7568(20)30014-3 PMC7721684

[16] Kalyani RR , Tian J , Xue QL *et al*. Hyperglycemia and incidence of frailty and lower extremity mobility limitations in older women. J Am Geriatr Soc 2012; 60: 1701–1707. 22882211 10.1111/j.1532-5415.2012.04099.x PMC4144067

[17] Zaslavsky O , Walker RL , Crane PK , Gray SL , Larson EB . Glucose levels and risk of frailty. J Gerontol A Biol Sci Med Sci 2016; 71: 1223–1229. 26933160 10.1093/gerona/glw024 PMC4978362

[18] Chao CT , Wang J , Huang JW , Chan DC , Chien KL . COhort of GEriatric nephrology in NTUH (COGENT) study group. Hypoglycemic episodes are associated with an increased risk of incident frailty among new onset diabetic patients. J Diabetes Complications 2020; 34: 107492. 31806427 10.1016/j.jdiacomp.2019.107492

[19] Murao Y , Ishikawa J , Tamura Y *et al*. Association between physical performance during sit‐to‐stand motion and frailty in older adults with cardiometabolic diseases: a cross‐sectional, longitudinal study. BMC Geriatr 2023; 23: 337. 37254047 10.1186/s12877-023-04011-z PMC10228424

[20] Oba K , Ishikawa J , Tamura Y *et al*. Serum GDF15 level is associated with muscle strength and lower extremity function in older patients with cardiometabolic disease. Geriatr Gerontol Int 2020; 20: 980–987. 32886834 10.1111/ggi.14021

[21] Oba K , Ishikawa J , Tamura Y *et al*. Serum growth differentiation factor 15 levels predict the incidence of frailty among patients with Cardiometabolic diseases. Gerontology 2024; 70: 517–525. 38286122 10.1159/000536150

[22] Tamura Y , Shimoji K , Ishikawa J *et al*. Association between white matter alterations on diffusion tensor imaging and incidence of frailty in older adults with cardiometabolic diseases. Front Aging Neurosci 2022; 14: 912972. 35966786 10.3389/fnagi.2022.912972 PMC9363893

[23] Veronese N , Cereda E , Stubbs B *et al*. Risk of cardiovascular disease morbidity and mortality in frail and pre‐frail older adults: results from a meta‐analysis and exploratory meta‐regression analysis. Ageing Res Rev 2017; 35: 63–73. 28143778 10.1016/j.arr.2017.01.003 PMC6047747

[24] Tamura Y , Shimoji K , Ishikawa J *et al*. Associations between sarcopenia and white matter alterations in older adults with diabetes mellitus: a diffusion tensor imagingstudy. J Diabetes Investig 2021; 12: 633–640. 10.1111/jdi.13379 PMC8015831 32750745

[25] Tamura Y , Shimoji K , Ishikawa J *et al*. Subclinical atherosclerosis, vascular risk factors, and white matter alterations in diffusion tensor imaging findings of older adults with Cardiometabolic diseases. Front Aging Neurosci 2021; 13: 712385. 34489681 10.3389/fnagi.2021.712385 PMC8417784

[26] Tamura Y , Omura T , Toyoshima K , Araki A . Nutrition Management in Older Adults with diabetes: a review on the importance of shifting prevention strategies from metabolic syndrome to frailty. Nutrients 2020; 12: 3367. 33139628 10.3390/nu12113367 PMC7693664

[27] Anagnostis P , Gkekas NK , Achilla C *et al*. Type 2 diabetes mellitus is associated with increased risk of sarcopenia: a systematic review and meta‐analysis. Calcif Tissue Int 2020; 107: 453–463. 32772138 10.1007/s00223-020-00742-y

[28] Ai Y , Xu R , Liu L . The prevalence and risk factors of sarcopenia in patients with type 2 diabetes mellitus: a systematic review and meta‐analysis. Diabetol Metab Syndr 2021; 13: 93. 34479652 10.1186/s13098-021-00707-7 PMC8414692

[29] Chuan F , Chen S , Ye X *et al*. Sarcopenic obesity predicts negative health outcomes among older patients with type 2 diabetes: the ageing and body composition of diabetes (ABCD) cohort study. Clin Nutr 2022; 41: 2740–2748. 36370663 10.1016/j.clnu.2022.10.023

[30] Fukuda T , Bouchi R , Takeuchi T *et al*. Sarcopenic obesity assessed using dual energy X‐ray absorptiometry (DXA) can predict cardiovascular disease in patients with type 2 diabetes: a retrospective observational study. Cardiovasc Diabetol 2018; 17: 55. 29636045 10.1186/s12933-018-0700-5 PMC5891961

[31] Hirani V , Naganathan V , Blyth F *et al*. Longitudinal associations between body composition, sarcopenic obesity and outcomes of frailty, disability, institutionalisation and mortality in community‐dwelling older men: the Concord health and ageing in men project. Age Ageing 2017; 46: 413–420. 27932368 10.1093/ageing/afw214

[32] Atkins JL , Whincup PH , Morris RW , Lennon LT , Papacosta O , Wannamethee SG . Sarcopenic obesity and risk of cardiovascular disease and mortality: a population‐based cohort study of older men. J Am Geriatr Soc 2014; 62: 253–260. 24428349 10.1111/jgs.12652 PMC4234002

[33] Tamura Y , Araki A . Diabetes mellitus and white matter hyperintensity. Geriatr Gerontol Int 2015; 15: 34–42. 10.1111/ggi.12666 26671155

[34] McAllister DA , Read SH , Kerssens J *et al*. Incidence of hospitalization for heart failure and case‐fatality among 3.25 million people with and without diabetes mellitus. Circulation 2018; 138: 2774–2786. 29950404 10.1161/CIRCULATIONAHA.118.034986 PMC6287897

[35] Kernick D , Chew‐Graham CA , O'Flynn N . Clinical assessment and management of multimorbidity: NICE guideline. Br J Gen Pract 2017; 67: 235–236. 28450343 10.3399/bjgp17X690857 PMC5409424

[36] Caughey GE , Roughead EE , Vitry AI , McDermott RA , Shakib S , Gilbert AL . Comorbidity in the elderly with diabetes: identification of areas of potential treatment conflicts. Diabetes Res Clin Pract 2010; 87: 385–393. 19923032 10.1016/j.diabres.2009.10.019

[37] Ohsugi M , Eiki JI , Iglay K , Tetsuka J , Tokita S , Ueki K . Comorbidities and complications in Japanese patients with type 2 diabetes mellitus: retrospective analyses of J‐DREAMS, an advanced electronic medical records database. Diabetes Res Clin Pract 2021; 178: 108845. 33933501 10.1016/j.diabres.2021.108845

[38] Chiang JI , Jani BD , Mair FS *et al*. Associations between multimorbidity, all‐cause mortality and glycaemia in people with type 2 diabetes: a systematic review. PLoS One 2018; 13: e0209585. 30586451 10.1371/journal.pone.0209585 PMC6306267

[39] Omura T , Tamura Y , Sakurai T *et al*. Functional categories based on cognition and activities of daily living predict all‐cause mortality in older adults with diabetes mellitus: the Japanese elderly diabetes intervention trial. Geriatr Gerontol Int 2021; 21: 512–518. 33890351 10.1111/ggi.14171

[40] Mazya AL , Garvin P , Ekdahl AW . Outpatient comprehensive geriatric assessment: effects on frailty and mortality in old people with multimorbidity and high health care utilization. Aging Clin Exp Res 2019; 31: 519–525. 30039453 10.1007/s40520-018-1004-z PMC6439176

[41] Rockwood K , Mitnitski A . Frailty in relation to the accumulation of deficits. J Gerontol A Biol Sci Med Sci 2007; 62: 722–727. 17634318 10.1093/gerona/62.7.722

[42] Kojima G , Iliffe S , Walters K . Frailty index as a predictor of mortality: a systematic review and meta‐analysis. Age Ageing 2018; 47: 193–200. 29040347 10.1093/ageing/afx162

[43] Simpson FR , Pajewski NM , Nicklas B *et al*. Impact of multidomain lifestyle intervention on frailty through the lens of deficit accumulation in adults with type 2 diabetes mellitus. J Gerontol A Biol Sci Med Sci 2020; 75: 1921–1927. 31559418 10.1093/gerona/glz197 PMC7518562

[44] Sanz París A , García JM , Gómez‐Candela C *et al*. Malnutrition prevalence in hospitalized elderly diabetic patients. Nutr Hosp 2013; 28: 592–599. 23848076 10.3305/nh.2013.28.3.6472

[45] Alfonso‐Rosa RM , Del Pozo‐Cruz B , Del Pozo‐Cruz J , Del Pozo‐Cruz JT , Sañudo B . The relationship between nutritional status, functional capacity, and health‐related quality of life in older adults with type 2 diabetes: a pilot explanatory study. J Nutr Health Aging 2013; 17: 315–321.23538652 10.1007/s12603-013-0028-5

[46] Araki E , Goto A , Kondo T *et al*. Japanese clinical practice guideline for diabetes 2019. Diabetol Int. 2020; 11: 173–177. 10.1007/s13340-020-00439-5 PMC7387396 32802702

[47] Omura T , Tamura Y , Yamaoka T *et al*. Assessing the association between optimal energy intake and all‐cause mortality in older patients with diabetes mellitus using the Japanese elderly diabetes intervention trial. Geriatr Gerontol Int 2020; 20: 59–65. 31820841 10.1111/ggi.13820 PMC7003876

[48] Volkert D , Beck AM , Cederholm T *et al*. ESPEN guideline on clinical nutrition and hydration in geriatrics. Clin Nutr 2019; 38: 10–47. 30005900 10.1016/j.clnu.2018.05.024

[49] Rahi B , Morais JA , Gaudreau P , Payette H , Shatenstein B . Energy and protein intakes and their association with a decline in functional capacity among diabetic older adults from the NuAge cohort. Eur J Nutr 2016; 55: 1729–1739. 26179475 10.1007/s00394-015-0991-1

[50] Yamaoka T , Araki A , Tamura Y *et al*. Association between low protein intake and mortality in patients with type 2 diabetes. Nutrients 2020; 12: 1629. 32492838 10.3390/nu12061629 PMC7352318

[51] Kumagai S , Watanabe S , Shibata H *et al*. Effects of dietary variety on declines in high‐level functional capacity in elderly people living in a community. Nihon Koshu Eisei Zasshi 2003; 50: 1117–1124. 14750363

[52] Hayakawa M , Motokawa K , Mikami Y *et al*. Low dietary variety and diabetes mellitus are associated with frailty among community‐dwelling older Japanese adults: a cross‐sectional study. Nutrients 2021; 13: 641. 33669388 10.3390/nu13020641 PMC7920314

[53] Espeland MA , Lipska K , Miller ME *et al*. Effects of physical activity intervention on physical and cognitive function in sedentary adults with and without diabetes. J Gerontol A Biol Sci Med Sci 2017; 72: 861–866. 27590629 10.1093/gerona/glw179 PMC6075086

[54] Rodriguez‐Mañas L , Laosa O , Vellas B *et al*. Effectiveness of a multimodal intervention in functionally impaired older people with type 2 diabetes mellitus. J Cachexia Sarcopenia Muscle 2019; 10: 721–733. 31016897 10.1002/jcsm.12432 PMC6711410

[55] Geller AI , Shehab N , Lovegrove MC *et al*. National estimates of insulin‐related hypoglycemia and errors leading to emergency department visits and hospitalizations. JAMA Intern Med 2014; 174: 678–686. 24615164 10.1001/jamainternmed.2014.136 PMC4631022

[56] Yaffe K , Falvey CM , Hamilton N *et al*. Association between hypoglycemia and dementia in a biracial cohort of older adults with diabetes mellitus. JAMA Intern Med 2013; 173: 1300–1306. 23753199 10.1001/jamainternmed.2013.6176 PMC4041621

[57] Feil DG , Rajan M , Soroka O , Tseng CL , Miller DR , Pogach LM . Risk of hypoglycemia in older veterans with dementia and cognitive impairment: implications for practice and policy. J Am Geriatr Soc 2011; 59: 2263–2272. 22150156 10.1111/j.1532-5415.2011.03726.x

[58] Lee AK , Lee CJ , Huang ES , Sharrett AR , Coresh J , Selvin E . Risk factors for severe hypoglycemia in black and white adults with diabetes: the atherosclerosis risk in communities (ARIC) study. Diabetes Care 2017; 40: 1661–1667. 28928117 10.2337/dc17-0819 PMC5711330

[59] Katon WJ , Young BA , Russo J *et al*. Association of depression with increased risk of severe hypoglycemic episodes in patients with diabetes. Ann Fam Med 2013; 11: 245–250. 23690324 10.1370/afm.1501 PMC3659141

[60] Schloot NC , Haupt A , Schütt M *et al*. Risk of severe hypoglycemia in sulfonylurea‐treated patients from diabetes centers in Germany/Austria: how big is the problem? Which patients are at risk? Diabetes Metab Res Rev 2016; 32: 316–324. 26409039 10.1002/dmrr.2722

[61] Shorr RI , Ray WA , Daugherty JR , Griffin M . Incidence and risk factors for serious hypoglycemia in older persons using insulin or sulfonylureas. Arch Intern Med 1997; 157: 1681–1686. 9250229

[62] ALEissa MS , AlGhofaili IA , Alotaibe HF *et al*. Incidence and risk factors associated with hypoglycemia among patients with chronic kidney disease: a systematic review. J Family Community Med 2020; 27: 157–162. 33354145 10.4103/jfcm.JFCM_304_19 PMC7745784

[63] Lin YY , Hsu CW , Sheu WH , Chu SJ , Wu CP , Tsai SH . Risk factors for recurrent hypoglycemia in hospitalized diabetic patients admitted for severe hypoglycemia. Yonsei Med J 2010; 51: 367–374. 20376889 10.3349/ymj.2010.51.3.367 PMC2852792

[64] Bramlage P , Gitt AK , Binz C , Krekler M , Deeg E , Tschöpe D . Oral antidiabetic treatment in type‐2 diabetes in the elderly: balancing the need for glucose control and the risk of hypoglycemia. Cardiovasc Diabetol 2012; 11: 122. 23039216 10.1186/1475-2840-11-122 PMC3508810

[65] Toschi E , Slyne C , Sifre K *et al*. The relationship between CGM‐derived metrics, A1C, and risk of hypoglycemia in older adults with type 1 diabetes. Diabetes Care 2020; 43: 2349–2354. 32461211 10.2337/dc20-0016 PMC7510030

[66] Schopman JE , Simon AC , Hoefnagel SJ , Hoekstra JB , Scholten RJ , Holleman F . The incidence of mild and severe hypoglycaemia in patients with type 2 diabetes mellitus treated with sulfonylureas: a systematic review and meta‐analysis. Diabetes Metab Res Rev 2014; 30: 11–22. 24030920 10.1002/dmrr.2470

[67] Andersen SE , Christensen M . Hypoglycaemia when adding sulphonylurea to metformin: a systematic review and network meta‐analysis. Br J Clin Pharmacol 2016; 82: 1291–1302. 27426428 10.1111/bcp.13059 PMC5061791

[68] Fløde M , Hermann M , Haugstvedt A *et al*. High number of hypoglycaemic episodes identified by CGM among home‐dwelling older people with diabetes: an observational study in Norway. BMC Endocr Disord 2023; 23: 218. 37817166 10.1186/s12902-023-01472-6 PMC10566065

[69] Bristol AA , Litchman M , Berg C *et al*. Using continuous glucose monitoring and data sharing to encourage collaboration among older adults with type 1 diabetes and their care partners: qualitative descriptive study. JMIR Nurs 2023; 26: e46627. 10.2196/46627 PMC10413231 37494110

[70] Araki A . Treatment of diabetes mellitus in older patients. (in Japanese). J Clin Exp Med 2015; 252: 537–554.

[71] Gottlieb ER , Estiverne C , Tolan NV , Melanson SEF , Mendu ML . Estimated GFR with cystatin C and creatinine in clinical practice: a retrospective cohort study. Kidney Med 2023; 5: 100600. 36879723 10.1016/j.xkme.2023.100600 PMC9984886

[72] Orloff J , Min JY , Mushlin A , Flory J . Safety and effectiveness of metformin in patients with reduced renal function: a systematic review. Diabetes Obes Metab 2021; 23: 2035–2047. 34009711 10.1111/dom.14440

[73] Munshi MN , Slyne C , Segal AR , Saul N , Lyons C , Weinger K . Simplification of insulin regimen in older adults and risk of hypoglycemia. JAMA Intern Med 2016; 176: 1023–1025. 27273335 10.1001/jamainternmed.2016.2288

[74] Giugliano D , Longo M , Caruso P *et al*. Feasibility of simplification from a basal‐bolus insulin regimen to a fixed‐ratio formulation of basal insulin plus a GLP‐1RA or to basal insulin plus an SGLT2 inhibitor:BEYOND, a randomized. Pragmatic Trial Diabetes Care 2021; 44: 1353–1360. 33883195 10.2337/dc20-2623 PMC8247516

[75] Bajaj HS , Aberle J , Davies M *et al*. Once‐weekly insulin Icodec with dosing guide app versus once‐daily basal insulin analogues in insulin‐naive type 2 diabetes (ONWARDS 5): a randomized trial. Ann Intern Med 2023; 176: 1476–1485. 37748181 10.7326/M23-1288

[76] Frias J , Chien J , Zhang Q *et al*. Safety and efficacy of once‐weekly basal insulin fc in people with type 2 diabetes previously treated with basal insulin: a multicentre, open‐label, randomised, phase 2 study. Lancet Diabetes Endocrinol 2023; 11: 158–168. 36758572 10.1016/S2213-8587(22)00388-6

[77] Karagiannis T , Tsapas A , Athanasiadou E *et al*. GLP‐1 receptor agonists and SGLT2 inhibitors for older people with type 2 diabetes: a systematic review and meta‐analysis. Diabetes Res Clin Pract 2021; 174: 108737. 33705820 10.1016/j.diabres.2021.108737

[78] Nakai M , Iwanaga Y , Kanaoka K *et al*. Contemporary use of SGLT2 inhibitors in heart failure patients with diabetes mellitus: a comparison of DPP4 inhibitors in a nationwide electric health database of the superaged society. Cardiovasc Diabetol 2022; 21: 157. 35964039 10.1186/s12933-022-01586-6 PMC9375946

[79] Kutz A , Kim DH , Wexler DJ *et al*. Comparative cardiovascular effectiveness and safety of SGLT‐2 inhibitors, GLP‐1 receptor agonists, and DPP‐4 inhibitors according to frailty in type 2 diabetes. Diabetes Care 2023; 46: 2004–2014. 37677118 10.2337/dc23-0671 PMC10620535

[80] Tang H , Shao H , Shaaban CE *et al*. Newer glucose‐lowering drugs and risk of dementia: a systematic review and meta‐analysis of observational studies. J Am Geriatr Soc 2023; 71: 2096–2106. 36821780 10.1111/jgs.18306 PMC10363181

[81] Nishioka Y , Noda T , Okada S *et al*. Incidence and seasonality of type 1 diabetes: a population‐based 3‐year cohort study using the National Database in Japan. BMJ Open Diabetes Res Care 2020; 8: e001262. 10.1136/bmjdrc-2020-001262 PMC7526280 32994226

[82] Takada S , Hirokazu H , Yamagishi K , Hideki S , Masayuki E . Predictors of the onset of type 1 diabetes obtained from real‐world data analysis in cancer patients treated with immune checkpoint inhibitors. Asian Pac J Cancer Prev 2020; 21: 1697–1699. 32592366 10.31557/APJCP.2020.21.6.1697 PMC7568867

[83] Abiru N , Shimada A , Nishimura R , Matsuhisa M , Ozaki A , Ikegami H . Glycemic control status, diabetes management patterns, and clinical characteristics of adults with type 1 diabetes in Japan: study of Adults' Glycemia in T1DM subanalysis. Diabetol Int 2021; 12: 460–473. 34567927 10.1007/s13340-021-00504-7 PMC8413413

[84] Miller KM , Kanapka LG , Rickels MR *et al*. Benefit of continuous glucose monitoring in reducing hypoglycemia is sustained through 12 months of use among older adults with type 1 diabetes. Diabetes Technol Ther 2022; 24: 424–434. 35294272 10.1089/dia.2021.0503 PMC9208859

[85] Munshi M , Slyne C , Davis D *et al*. Use of Technology in Older Adults with type 1 diabetes: clinical characteristics and glycemic metrics. Diabetes Technol Ther 2022; 24: 1–9. 34524033 10.1089/dia.2021.0246 PMC8783629

[86] Committee report: Glycemic targets for elderly patients with diabetes: Japan Diabetes Society (JDS)/Japan Geriatrics Society (JGS) Joint committee on improving care for elderly patients with diabetes . Glycemic targets for elderly patients with diabetes. J Diabetes Investig 2017; 8: 126–128. 10.1111/jdi.12599 PMC5217949 28054465

[87] LeRoith D , Biessels GJ , Braithwaite SS *et al*. Treatment of diabetes in older adults: an Endocrine Society* clinical practice guideline. J Clin Endocrinol Metab 2019; 104: 1520–1574. 30903688 10.1210/jc.2019-00198 PMC7271968

[88] American Diabetes Association Professional Practice Committee . 13. Older adults: standards of Care in Diabetes‐2024. Diabetes Care 2024; 47: S244–S257. 38078580 10.2337/dc24-S013 PMC10725804

[89] Conlin PR , Colburn J , Aron D , Pries RM , Tschanz MP , Pogach L . Synopsis of the 2017 U.S. Department of Veterans Affairs/U.S. Department of Defense clinical practice guideline: Management of Type 2 diabetes mellitus. Ann Intern Med 2017; 167: 655–663. 29059687 10.7326/M17-1362

[90] Hatano Y , Araki A , Matsumoto M , Ishibashi S . Low hemoglobin A1c and low body mass index are associated with dementia and activities of daily living disability among Japanese nursing home residents with diabetes. Geriatr Gerontol Int 2019; 19: 854–860. 31257696 10.1111/ggi.13728

[91] Sugimoto T , Ono R , Kimura A *et al*. Impact of glycemic control on daily living activities over 1‐year follow‐up in memory clinic patients with diabetes. J Am Med Dir Assoc 2019; 20: 792–794. 31043357 10.1016/j.jamda.2019.03.008

[92] Sugimoto T , Sakurai T , Uchida K *et al*. Impact of type 2 diabetes and glycated hemoglobin levels within the recommended target range on mortality in older adults with cognitive impairment receiving Care at a Memory Clinic: NCGG‐STORIES. Diabetes Care 2024; 47: 864–872. 38470970 10.2337/dc23-2324

[93] Conlin PR , Zhang L , Li D , Nelson RE , Prentice JC , Mohr DC . Association of hemoglobin A1c stability with mortality and diabetes complications in older adults with diabetes. BMJ Open Diabetes Res Care 2023; 11: e003211. 10.1136/bmjdrc-2022-003211 PMC10083809 37024152

[94] Lipska KJ , Huang ES , Liu JY *et al*. Glycemic control and diabetes complications across health status categories in older adults treated with insulin or insulin secretagogues: the Diabetes & Aging Study. J Am Geriatr Soc 2023; 71: 3692–3700. 37638777 10.1111/jgs.18565 PMC10872822

[95] Toyoshima K , Araki A , Tamura Y *et al*. Development of the dementia assessment sheet for community‐based integrated care system 8‐items, a short version of the dementia assessment sheet for community‐based integrated care system 21‐items, for the assessment of cognitive and daily functions. Geriatr Gerontol Int 2018; 18: 1458–1462. 30225857 10.1111/ggi.13512

[96] Toyoshima K , Araki A , Tamura Y *et al*. Use of dementia assessment sheet for community‐based integrated care system 8‐items (DASC‐8) for the screening of frailty and components of comprehensive geriatric assessment. Geriatr Gerontol Int 2020; 20: 1157–1163. 33067921 10.1111/ggi.14057

[97] Katsumata Y , Toyoshima K , Tamura Y *et al*. Categorization using the dementia assessment sheet for community‐based integrated care system 8‐items (DASC‐8) based on cognitive function and activities of daily living predicts frailty, disability and mortality in older adults. Geriatr Gerontol Int 2024; suppl 1: 150–155. 10.1111/ggi.14715 37872859

[98] Ellis G , Gardner M , Tsiachristas A *et al*. Comprehensive geriatric assessment for older adults admitted to hospital. Cochrane Database Syst Rev 2017; 9: CD006211. 28898390 10.1002/14651858.CD006211.pub3 PMC6484374

[99] Munshi MN , Segal AR , Suhl E *et al*. Assessment of barriers to improve diabetes management in older adults: a randomized controlled study. Diabetes Care 2013; 36: 543–549.[Correction added on 30 October 2024, after first online publication: Reference citation 90 in the first paragraph of the section ‘Individualized treatment based on categorization and CGA’ has been corrected to 96. References 83 and 84 in the References list have also been re‐ordered.] 23193208 10.2337/dc12-1303 PMC3579376

